# Unresolved advantages of multipartitism in spatially structured environments

**DOI:** 10.1093/ve/veab004

**Published:** 2021-01-25

**Authors:** Mark P Zwart, Stéphane Blanc, Marcelle Johnson, Susanna Manrubia, Yannis Michalakis, Mircea T Sofonea

**Affiliations:** 1 Department of Microbial Ecology, Netherlands Institute of Ecology (NIOO-KNAW), Postbus 50, Wageningen 6700 AB, The Netherlands; 2 BGPI, INRA, CIRAD, Montpellier SupAgro, Univ Montpellier, Montpellier 34398, France; 3 National Centre for Biotechnology (CSIC), C/Darwin no 3, Campus de Cantoblanco, Madrid 28049, Spain; 4 UMR MIVEGEC 5290, Université de Montpellier-CNRS-IRD, Montpellier 34394, France; 5 Centre of Research in Ecology and Evolution of Diseases (CREES), Montpellier 34394, France

## Abstract

Multipartite viruses have segmented genomes and package each of their genome segments individually into distinct virus particles. Multipartitism is common among plant viruses, but why this apparently costly genome organization and packaging has evolved remains unclear. Recently Zhang and colleagues developed network epidemiology models to study the epidemic spread of multipartite viruses and their distribution over plant and animal hosts (*Phys. Rev. Lett.* 2019, 123, 138101). In this short commentary, we call into question the relevance of these results because of key model assumptions. First, the model of plant hosts assumes virus transmission only occurs between adjacent plants. This assumption overlooks the basic but imperative fact that most multipartite viruses are transmitted over variable distances by mobile animal vectors, rendering the model results irrelevant to differences between plant and animal hosts. Second, when not all genome segments of a multipartite virus are transmitted to a host, the model assumes an incessant latent infection occurs. This is a bold assumption for which there is no evidence to date, making the relevance of these results to understanding multipartitism questionable.

## 1. Introduction

Multipartite viruses have segmented genomes and package these genome segments into multiple virus particles (Fulton 1962; Sicard et al. 2016). Viruses are often subject to transmission bottlenecks during their spread within or between hosts and, as segments then can be lost, multipartitism appears to come at a considerable cost to transmission (Iranzo and Manrubia 2012; Sánchez-Navarro, Zwart, and Elena 2013; Gutiérrez and Zwart 2018). Multipartite viruses are nevertheless quite common (Sicard et al. 2016; Lucía-Sanz and Manrubia 2017), raising the question why such a potentially costly feature has arisen and what benefits may be associated with it. One important observation that also calls for an explanation is that multipartite viruses commonly infect plants and fungi, but only a small number are known to infect animals (Ladner et al. 2016; Sicard et al. 2016).

Traditionally, the advantages of multipartitism have been sought in their molecular structure. Early proposals regarded reassortment—that is the exchange of entire genome segments between two distinct viral genotypes—as the possible key advantage for multipartite viruses (Reanney 1982), which were initially believed to be only RNA viruses. At that time, recombination was considered impossible in RNA and thus genome segmentation and reassortment have been proposed as a substitute for genetic exchange. That hypothesis was soon dismissed, however, when recombination between RNA molecules and multipartite viruses with DNA genomes was discovered. It has been put forward that the lower mutational load that should affect shorter genomes (Chao 1991) or a concomitant increase in the replication rate (Nee 1987) could also be potential advantages. But none of these suggestions have received empirical support so far (Sicard et al. 2016) and, while they could account for genome segmentation, none of them can explain the separate encapsidation of segments. Currently, a sound conjecture under empirical exploration states that the host-dependent variable frequency of the segments might ensure fast adaptation through differences in copy number of genes, with an effect on gene expression (Sicard et al. 2013; Sicard et al. 2019; Michalakis and Blanc 2020). This last hypothesis links molecular advantages to ecological ones, since it involves fast adaptation to different hosts—which implicitly assumes that multipartite viral forms would be more generalist, and therefore have a larger diversity of hosts under reach due to their architecture, than monopartite or segmented species. A consonant proposal is that multipartite viruses might represent opportunistic associations (in evolutionary time) that allow fast adaptation to available ecological niches (Lucía-Sanz and Manrubia 2017).

The observation that multipartite viruses preferentially infect plants has prompted the investigation of how the network of contacts between hosts affects their propagation (Valdano et al. 2019). In a recent publication (Zhang et al. 2019), a compartmental epidemiological model is linked to propagation in spatially structured host arrays to delve into the ecological advantages of multipartitism. This comment discusses certain essential assumptions to derive that model and the subsequent advantage of multipartite viruses that, in the light of our current understanding of these viruses, are not justified.

## 2. Epidemiology of multipartite viruses in a spatially explicit model

In Zhang et al. (2019), a network epidemiology approach is used in an effort to explain the high occurrence of multipartite viruses on plants as hosts. The authors developed an SLIR (S: susceptible; L: latent; I: infectious and R: removed) epidemic model to study virus epidemics. Here, we highlight two salient features of this model that are essential to derive the results reported and heavily impinge on its biological relevance. First, the authors contrast two different interaction structures for the hosts. A static interaction structure is included in which the connections between hosts are fixed over time. The authors argue this network structure is representative of plant hosts, stating that as they are sessile their interaction structures change much more slowly than infection spreads. The authors also include an annealed interaction structure in which the network is rearranged randomly at each time step, arguing this network structure might better represent animal hosts. Second, the authors include an L state in their models to represent a condition in which the host has been infected with some but not all genome segments of the multipartite virus, and therefore is not yet infectious. They do not include the identities of different segments in their model but simply assume that each subsequent virus transmission event randomly confers a new (or a few new) virus genome segment(s), occasionally the whole set of them. There might be a hierarchy of L states with increasing number of segments, but once a segment is acquired, the host remains in that state until it has gathered the whole set of segments, transitioning to the I state at that point.

In the framework of the SLIR model, with the assumptions stated, the authors find that a multipartite virus is more likely to persist under a static contact network than an annealed network if the average number of contacts per individual (the average degree of the network) is above a threshold that they estimate. This result is to be expected given the model assumptions. An infectious host is more likely to transmit multiple viral segments to the same susceptible host in a static rather than an annealed interaction structure, since in the static structure the transmission of various segments between a specific S–I or L–I pair can happen at multiple time points. In an annealed network, the transmission of segments is affected by a dilution process that prevents the formation of clusters of infected hosts, which are the only ones that effectively produce and transmit segments to other hosts in susceptible or latent states. As the authors assume the static interaction structure is representative for the transmission of plant diseases, they conclude that this difference may explain the distribution of multipartite viruses over hosts. This result is based on an untenable assertion about plant–virus transmission and on the existence of an L state that, to date, does not have empirical support. Since these two assumptions qualitatively affect the results attained, it is unclear how this article enhances our understanding of the between-host spread of multipartite viruses. Finally, as the study concerns the distribution of multipartite viruses over plants and animals, it does not suffice to study the epidemic behavior of different viruses in isolation. Below we detail each of these three important criticisms.

## 3. Unjustified assumptions of an SLIR model for multipartite virus propagation

First, the assumption that virus-transmission patterns between plants will be captured by a static network (Zhang et al. 2019) cannot be supported. Although plants are sessile, the vast majority of plant–virus transmission is due to vectors (which mostly consist of insects such as aphids), and this also holds for the multipartite viruses (Whitfield et al. 2015; Lucía-Sanz and Manrubia 2017). These insects can move different distances between plants, including long-range flights, and their movements are not stereotyped (e.g. vector movement often depends on subtle cues and can therefore be highly variable) (Carter 1961; Kring 1972; Hooks and Fereres 2006). Therefore, even as a first approximation, a static network scenario fails to capture the nature of the between-host contact network for plant–virus spread.

Second, the study results are based on an assumption that is insufficiently substantiated and not discussed: the existence of L states. The authors propose that transmission of one virus segment changes the host state, so that it can become infectious if the other virus segment(s) is transmitted later on (Zhang et al. 2019). Epidemiological models often consider a state called E, characteristic of susceptible-exposed-infectious-removed models. This state is included in epidemiological models (Tang et al. 2020) to consider either that viral infection of a host does not immediately make the host infectious for other individuals or that individuals might be infectious but asymptomatic. The consideration of state E does not change the phenomenological behavior of the more fundamental SIR model but can account for a delay in symptoms onset or for a modification of the effective infectivity in the system. E states, by definition, cannot revert to S states, since the natural progression of infected individuals is to state R (which typically includes both recovered—and usually immune—and dead individuals). However, the interpretation of the L state (Zhang et al. 2019) is very different, because the biological processes underlying this state do not represent an irreversible, natural progression. For the vast majority of multipartite viruses, the genes that code for the essential components of a successful infection are present on different genome segments (Fulton 1962; Sicard et al. 2016). When any essential component is missing, the virus cannot replicate or spread within that host individual and thus cannot be subsequently transmitted (Taschner et al. 1991; Sánchez-Navarro, Zwart, and Elena 2013; Sicard et al. 2016; Grigoras et al. 2018). Whether and for how long individual segments can persist in the host when the full complement of the other segments is absent has not been investigated yet, and so its potential relevance is totally unknown. The simple degradation of viral nucleic acids, thus of the ‘waiting’ incomplete set of segments, could result in reversions from state L to S. To compound this issue, the model by Zhang et al. (2019) assumes that L states can only accumulate segments, and L states will therefore exist indefinitely or progress to I. However, if we hypothesize an L state does exist, it would probably be transient, reverting to S after a period of unknown but, certainly, not unlimited duration. This reversion can significantly affect the results reported and, importantly, the threshold value calculated for the average number of neighbors over which the advantage of multipartite viral forms shows up: any process diminishing the effectiveness of contagion (and reversion to S is one such process) will hinder those advantages and therefore require a higher threshold.

Is there any evidence L states might exist? It was recently shown that nanoviruses have distributed replication at the cellular level (Sicard et al. 2019). Within a virus-infected plant, many virus-infected cells do not contain the full complement of genome segments, while the gene products coded on these segments are present. Some multipartite viruses can therefore share gene products, but only between cells within an infected plant. Moreover, even though field isolates invariably contain all segments, in laboratory inoculations multipartite viruses with a high number of segments can replicate without their full complement of segments: for example, an octopartite nanovirus could lose up to three segments (Grigoras et al. 2018). However, in this case, a core of five specific segments is always present (Grigoras et al. 2018). Incomplete infections that nevertheless autonomously replicate and transmit should be considered I states, and not L states. Moreover, whether incomplete infections that cannot autonomously accomplish the lifecycle can be complemented, and hence potentially represent L states, and within which time window requires further investigation as ‘superinfection exclusion’, common among viruses (Folimonova 2012; Kumar et al. 2018), could impede complementation. Further, in multipartite viruses, the different segments are never equivalent with respect to replication and other viral functions. Hence, an appropriate consideration of complementation would require tracking the identity of segments, a feature currently lacking in the model (Zhang et al. 2019), and not just compounding them into classes based on the number of segments. The empirical observation of an L state would astound most virologists and, in the light of the above, cannot be taken for granted.

Third, the study focuses on which contact network, static or annealed, is more amenable to multipartite virus epidemics (Zhang et al. 2019). As stated previously, the transmission network of the vast majority of plant viruses cannot be considered as static because they are vectored by animals. Moreover, the average number of contacts required in the framework of the SLIR model for the static scenario to be advantageous (as compared to the annealed scenario) exceeds by far what could be considered nearest neighbors of an infected individual. Actually, if the average number of contacts is small (say 4 or 8, as in typical diffusive processes), multipartite forms do not propagate more efficiently in static than in annealed settings. The competition between monopartite and multipartite forms was never considered in the SLIR framework even if, given that the two architectures are present in multiple viral families (Sicard et al. 2016; Lucía-Sanz and Manrubia 2017), a relevant question from an evolutionary and adaptive viewpoint is why the successful architecture in certain hosts is the multipartite one, and not a monopartite counterpart. In that context, it would be important to identify conditions favoring the evolution of multipartite viruses, by considering when they can outcompete monopartite viruses (Iranzo and Manrubia 2012; Gallet et al. 2018; Valdano et al. 2019). Without considering competition, one cannot draw conclusions on the advantage of being multipartite.

## 4. Final remarks

The intriguing phenomenology of the model, explosive transitions to endemicity (Fig. 1 in Zhang et al. 2019) and lower epidemic threshold values (Fig. 2 in Zhang et al. 2019) for multipartite viruses in static networks, only occur for the SLIR and not the SIR model (Zhang et al. 2019). Similar processes are of relevance in physics, as transitions to synchronization in networks of oscillators that occur in a sudden, explosive manner (Boccaletti et al. 2006), and that may inspire the search for analogous behavior in biological systems. However, this sound but counterintuitive phenomenology relies on a clustered structure that has not yet found a counterpart in the macroecology of multipartite viral species, and that would be necessarily mediated by the L state. Hence, if and until evidence for the existence of a latent stage is found, it will remain unclear whether this model sheds any light on the transmission of multipartite viruses. Moreover, model assumptions on the interaction structure of hosts fundamentally clash with reality: because mobile vectors typically transmit plant viruses, a static network cannot approximate the interaction structure for plant virus diseases. Therefore, we feel the results reported in Zhang et al. (2019) will never have any explanatory power for the distribution of multipartite viruses over plant, fungal and animal hosts, regardless of whether our understanding of L states might change in the future.

## Funding

M.P.Z and M.L.J. were supported by a VIDI grant from The Netherlands Organisation for Scientific Research (NWO 016.Vidi.171.061) to M.P.Z.

Conflict of interest: None declared.

## References

[veab004-B1] Boccaletti S et al (2006) ‘Complex Networks: Structure and Dynamics’, Physics Reports, 424: 175–308.

[veab004-B2] Carter W (1961) ‘Ecological Aspects of Plant Virus Transmissions’, Annual Review of Entomology, 6: 347–70. 10.1146/annurev.en.06.010161.002023 13691158

[veab004-B3] Chao L (1991) ‘Levels of Selection, Evolution of Sex in RNA Viruses, and the Origin of Life’, Journal of Theoretical Biology, 153: 229–46. 1787738 10.1016/s0022-5193(05)80424-2

[veab004-B4] Folimonova S. Y (2012) ‘Superinfection Exclusion is an Active Virus-Controlled Function That Requires a Specific Viral Protein’, Journal of Virology, 86: 5554–61. 22398285 10.1128/JVI.00310-12 PMC3347309

[veab004-B5] Fulton R. W (1962) ‘The Effect of Dilution on Necrotic Ringspot Virus Infectivity and the Enhancement of Infectivity by Noninfective Virus’, Virology, 18: 477–85. 13960255 10.1016/0042-6822(62)90038-7

[veab004-B6] Gallet R et al (2018) ‘Small Bottleneck Size in a Highly Multipartite Virus during a Complete Infection Cycle’, J. Virol, 92: e00139–18. 29720515 10.1128/JVI.00139-18 PMC6026758

[veab004-B7] Grigoras I et al (2018) ‘Nanovirus DNA-N Encodes a Protein Mandatory for Aphid Transmission’, Virology, 522: 281–91. 30071404 10.1016/j.virol.2018.07.001

[veab004-B8] Gutiérrez S. , Zwart M. P (2018) ‘Population Bottlenecks in Multicomponent Viruses: First Forays into the Uncharted Territory of Genome-Formula Drift’, Current Opinion in Virology, 33: 184–90. 30471620 10.1016/j.coviro.2018.09.001

[veab004-B9] Hooks C. R. R. , Fereres A (2006) ‘Protecting Crops from Non-Persistently Aphid-Transmitted Viruses: A Review on the Use of Barrier Plants as a Management Tool’, Virus Research, 120: 1–16. 16780985 10.1016/j.virusres.2006.02.006

[veab004-B10] Iranzo J. , Manrubia S. C (2012) ‘Evolutionary Dynamics of Genome Segmentation in Multipartite Viruses’, Proceedings of the Royal Society B: Biological Sciences, 279: 3812–9. 10.1098/rspb.2012.1086 PMC3415918 22764164

[veab004-B11] Kring J. B (1972) ‘Flight Behavior of Aphids’, Annual Review of Entomology, 17: 461–92.

[veab004-B12] Kumar N et al (2018) ‘Virological and Immunological Outcomes of Coinfections’, Clin. Microbiol. Rev, 31: e00111–17. 29976554 10.1128/CMR.00111-17 PMC6148187

[veab004-B13] Ladner J. T et al (2016) ‘A Multicomponent Animal Virus Isolated from Mosquitoes’, Cell Host Microbe, 20: 1–11. 27569558 10.1016/j.chom.2016.07.011 PMC5025392

[veab004-B14] Lucía-Sanz A. , Manrubia S (2017) ‘Multipartite Viruses: Adaptive Trick or Evolutionary Treat?’, NPJ Systems Biology and Applications, 3: 34. 29263796 10.1038/s41540-017-0035-y PMC5680193

[veab004-B15] Michalakis Y. , Blanc S (2020) ‘The Curious Strategy of Multipartite Viruses’, Annual Review of Virology, 7: 203–18. 10.1146/annurev-virology-010220-063346 32991271

[veab004-B16] Nee S (1987) ‘The Evolution of Multicompartmental Genomes in Viruses’, Journal of Molecular Evolution, 25: 277–81. 3118044 10.1007/BF02603110

[veab004-B17] Reanney D. C (1982) ‘The Evolution of RNA Viruses’, Annual Review of Microbiology, 36: 47–73. 10.1146/annurev.mi.36.100182.000403 6756295

[veab004-B18] Sánchez-Navarro J. A. , Zwart M. P., Elena S. F (2013) ‘Effects of the Number of Genome Segments on Primary and Systemic Infections with a Multipartite Plant RNA Virus’, Journal of Virology, 87: 10805–15. 23903837 10.1128/JVI.01402-13 PMC3807391

[veab004-B19] Sicard A et al (2013) ‘Gene Copy Number is Differentially Regulated in a Multipartite Virus’, Nat. Commun, 4: 1–8. 10.1038/ncomms3248 23912259

[veab004-B20] Sicard A et al (2016) ‘The Strange Lifestyle of Multipartite Viruses’, PLoS Pathogens, 12: e1005819–19. 27812219 10.1371/journal.ppat.1005819 PMC5094692

[veab004-B21] Sicard A et al (2019) ‘A Multicellular Way of Life for a Multipartite Virus’, eLife, 8:10.7554/eLife.43599 PMC6414197 30857590

[veab004-B22] Tang L et al (2020) ‘A Review of Multi-Compartment Infectious Disease Models’, International Statistical Review , 88: 462–513. 32834402 10.1111/insr.12402 PMC7436714

[veab004-B23] Taschner P. E. M et al (1991) ‘Replication of an Incomplete Alfalfa Mosaic Virus Genome in Plants Transformed with Viral Replicase Genes’, Virology, 181: 445–50. 2014633 10.1016/0042-6822(91)90876-d

[veab004-B24] Valdano E et al (2019) ‘Endemicity and Prevalence of Multipartite Viruses under Heterogeneous between-Host Transmission’, PLOS Computational Biology, 15: e1006876. 30883545 10.1371/journal.pcbi.1006876 PMC6438571

[veab004-B25] Whitfield A. E. , Falk B. W., Rotenberg D (2015) ‘Insect Vector-Mediated Transmission of Plant Viruses’, Virology, 479-480: 278–89. 25824478 10.1016/j.virol.2015.03.026

[veab004-B26] Zhang Y et al (2019) ‘Advantage of Being Multicomponent and Spatial : Multipartite Viruses Colonize Structured Populations with Lower Thresholds’, Phys. Rev. Lett, 123: 138101. 31697512 10.1103/PhysRevLett.123.138101

